# Editorial: Reviews in receptor-receptor interactions as novel targets for drug development

**DOI:** 10.3389/fendo.2023.1185190

**Published:** 2023-03-24

**Authors:** Diego Guidolin, Manuela Marcoli, Amina S. Woods

**Affiliations:** ^1^ Department of Neuroscience, Section of Anatomy, University of Padova, Padova, Italy; ^2^ Department of Pharmacy, Section of Pharmacology and Toxicology, University of Genova, Genova, Italy; ^3^ Department of Pharmacology and Molecular Sciences, Johns Hopkins University School of Medicine, Baltimore, MD, United States; ^4^ National Institute on Drug Abuse Intramural Research Program (NIDA IRP), National Institute of Health (NIH), Baltimore, MD, United States

**Keywords:** receptors, receptor-receptor interactions, receptor crosstalk, receptor complexes, allostery, oligomerization

The concept of “receptor” was proposed at the beginning of the 20th century to explain the selective effects of drugs on their target and suggested that the action of a drug involved the formation of specific complexes with molecular structures in the target cells, thereby eliciting a cell response. Presently more than 4% of the human genome encodes cell receptors, organized into different families, including matrix receptors (e.g., integrins), ligand-gated and voltage-gated ion channels, intracellular receptors (such as nuclear hormone receptors), enzyme-linked receptors (such as receptor tyrosine kinases) and G protein-coupled receptors (GPCRs) constituting the largest family in mammals (1). As cells express several receptor subtypes, signals are both spatially and time integrated in order to generate appropriate cellular response upon activation of multiple receptors, the disruption of which could be pathological. In the last decades. mechanisms regulating receptor crosstalk have been proposed, either at the level of the receptors themselves or at the level of their signaling pathways (2).

This Research Topic received five papers, including one research article and four reviews, where the authors address different aspects of the complex interaction between receptors and receptor signaling pathways, with a specific attention to the pharmacological and therapeutic strategies that can emerge from a deeper understanding of these mechanisms.

A main path through which receptors can interact is represented by functional mechanisms, such as sharing of signaling pathways, receptor transactivation, or synergistic regulation of signaling crossroads. Intracellular calcium levels, for instance, can be controlled by two signaling pathways triggered by the co-activation of two GPCRs coupled to G_q_ and G_i/0_ proteins respectively (2). A further example is provided by Wang et al. in a paper focused on the changes in hormones and their receptors induced by hypoxia in the adult rat pituitary. In this study, corticotropin-releasing hormone receptor 1 (CRHR1) is significantly upregulated during hypoxia, leading to a modulation of transcription factors regulating the expression of not only pituitary hormones but also of their receptors, an effect blocked by a CRHR1 specific antagonist (CP154,526). Since the crosstalk among receptors regulates the pituitary functions, the obtained results allowed the outlining of a network of interactions involving transcription factors, hormones, and receptors, where CRHR1 plays a key regulatory role in hypoxia. Evidence concerning a network of functional receptor interactions occurring in prostate cancer and involving estrogen receptors was reviewed by Ramirez-de-Arellano et al. The effect of estrogen on the prostate gland is mediated by two nuclear receptors (ERα and ERβ) and by a GPCR receptor (GPER). The upregulation of ER receptors during progression of prostate cancer can alter the homeostatic balance of the GPER transmembrane signaling cascade, that inhibits cancer cell growth, leading to increased neoplastic activity in estrogen-sensitive tissues. An overview of the complex network of interactions among hormonal sub-systems in the polycystic ovary syndrome has been proposed by Emanuel et al. who also provided a comprehensive summarizing ‘wiring’ diagram, potentially helpful for a better interpretation of diagnostics tests and for the development of therapeutic protocols. In this network, functional interactions between receptors may play a role, as suggested by experimental data indicating an increased activity of the androgen receptors and a corresponding reduced expression of progesterone receptors in hypothalamic GABA neurons. The proposed overview, therefore, may open further research perspectives in the field.

In the early 1980s, *in vitro* and *in vivo* experiments provided indirect evidence that structural receptor-receptor interactions (RRI) may also occur, leading to the formation at the cell membrane of multimeric receptor complexes characterized by a cooperative dynamic (see Guidolin et al. for a recent review). In all receptor families, oligomeric organization thus emerged as an efficient mechanism for tuning the functionality of receptor proteins, including those able to signal as monomers, such as GPCRs (3). In this context, the review paper by Ramirez-de-Arellano et al. indicated that the action of the estrogen receptor ERβ (exhibiting five different variants) may depend on the differential amounts of heterodimers between variants formed upon stimulation by a specific ligand. Ferré et al. reviewed the evidence conveying a significant role of dopamine D_4_ receptor in the dopaminergic and noradrenergic modulation of the frontal cortico-striatal pyramidal neurons, having implications for the moderation of constructs of impulsivity as personality traits. This function is largely based on heterodimerization of the D_4_ receptor with the adrenergic α_2A_ or dopamine D_2_ receptors. D_4_ is an interesting GPCR also in view of the large number of polymorphic variants in humans (4) and the report by Ferré et al. indicates that some of them can further modulate the pharmacological and functional properties of α_2A_-D_4_ and D_2_-D_4_ heterodimers. Thus, the analysis of the available data supports D_4_ receptors as therapeutic targets for attention-deficit hyperactivity disorder and other impulse disorders. The so-called “target problem”, a critical aspect of drug development in the therapy of neuropsychiatric diseases, involving the selection of a proper target based on the detection of the supposed structural and/or functional alterations in brain networks, was the focus of the discussion proposed by Marcoli et al. in their review article. In this context, receptor complexes represent a structure of particular interest to increase the selectivity of pharmacological treatments. On one side, their structure and function are modulated by a network of interactions they establish with cell membrane proteins and lipids, and with the extracellular matrix. This feature could open up new pharmacological approaches to modify their signaling. On the other hand, the search for receptor heteromers’ selective compounds would be of key importance to fully exploit their properties. In this respect, the development of bivalent ligands or the development of allosteric modulators acting on specific allosteric sites emerging on the receptor structure when the complex forms, appear to be very promising strategies.

## Author contributions

All authors listed have made a substantial, direct, and intellectual contribution to the work and approved it for publication.
